# Metabolic implications of hypoxia and pseudohypoxia in pheochromocytoma and paraganglioma

**DOI:** 10.1007/s00441-018-2801-6

**Published:** 2018-02-15

**Authors:** Katarina Kluckova, Daniel A. Tennant

**Affiliations:** Institute of Metabolism and Systems Research, College of Medical and Dental Sciences, University of Birmingham, Edgbaston, Birmingham, B15 2TT UK

**Keywords:** Hypoxia, Pseudohypoxia, Metabolism, ROS, SDH

## Abstract

Hypoxia is a critical driver of cancer pathogenesis, directly inducing malignant phenotypes such as epithelial-mesenchymal transition, stem cell-like characteristics and metabolic transformation. However, hypoxia-associated phenotypes are often observed in cancer in the absence of hypoxia, a phenotype known as pseudohypoxia, which is very well documented in specific tumour types, including in paraganglioma/pheochromocytoma (PPGL). Approximately 40% of the PPGL tumours carry a germ line mutation in one of a number of susceptibility genes of which those that are found in succinate dehydrogenase (*SDH*) or in von Hippel-Lindau (*VHL*) genes manifest a strong pseudohypoxic phenotype. Mutations in SDH are oncogenic, forming tumours in a select subset of tissues, but the cause for this remains elusive. Although elevated succinate levels lead to increase in hypoxia-like signalling, there are other phenotypes that are being increasingly recognised in *SDH*-mutated PPGL, such as DNA hypermethylation. Further, recently unveiled changes in metabolic re-wiring of SDH-deficient cells might help to decipher cancer related roles of SDH in the future. In this review, we will discuss the various implications that the malfunctioning SDH can have and its impact on cancer development.

## Introduction

In 2000, Hanahan and Weinberg introduced the concept of six universal hallmarks of cancer (Hanahan and Weinberg 2000), which were enhanced around 10 years later with a further two hallmarks, and two ‘enabling characteristics’ (Hanahan and Weinberg 2011). The hallmarks can be elicited as a result of mutations in oncogenes and tumour suppressor genes—indeed, this is what the focus of much research has been on to date. Interestingly, it has also been argued that most of the hallmarks can arise as a result of the metabolic microenvironment of the tumour and, in particular, the pervasive low oxygen (hypoxic) environment often observed in most solid tumours (Kroemer and Pouyssegur 2008; Wigerup et al. 2016).

Hypoxia is a critical driver of cancer pathogenesis, directly inducing malignant phenotypes such as epithelial-mesenchymal transition, stem cell-like characteristics, and metabolic transformation. However, these hypoxia-driven phenotypes are often observed in cancer in the absence of hypoxia—a phenotype known as pseudohypoxia. This term was originally coined in the early 90s, referring to hypoxia-like change in the metabolic phenotype of cells in diabetes (Williamson et al. 1993). Since then, the acquisition of pseudohypoxic phenotypes has been observed in a number of pathological and physiological processes, including inflammation (Halligan et al. 2016), differentiation (Mohlin et al. 2017) and aging (Verdin 2015). The role of pseudohypoxia in tumourigenesis is well documented in specific tumour types, including in paraganglioma/pheochromocytoma (PPGL). In this review, we first briefly describe the PPGL tumours and general hypoxic signalling. Later, we focus on hypoxia/pseudohypoxia, various pathways leading to this condition and their relevance in the PPGL syndrome.

## Paraganglioma and pheochromocytoma tumours

PPGL are tumours of neuroendocrine origin and can arise at various sites of the body either of parasympathetic or sympathetic lineage-derived cells. Approximately 40% of the PPGL tumours carry a germ line mutation in one of a number of susceptibility genes, which makes PPGL the cancer syndrome with the highest reported degree of heritability (Dahia 2014). More recently, apparently sporadic cases were shown to harbour somatic mutations in known susceptibility genes (and some newly defined genes) which raises the number of a genetic driver event in these cancers to up to 70–80% (Burnichon et al. 2016; Curras-Freixes et al. 2017).

**Fig. 1:**
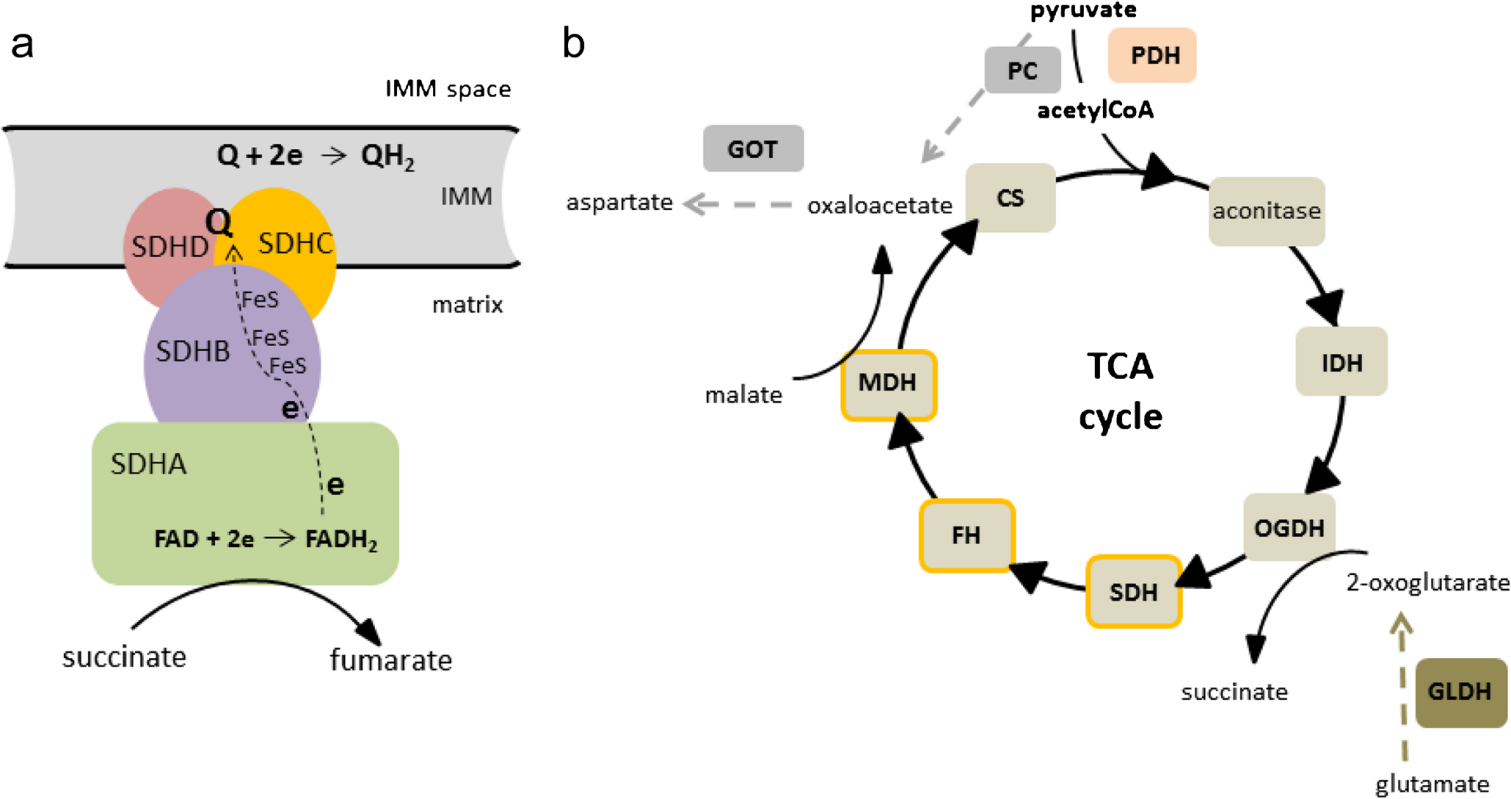
SDH structure and its role in TCA cycle. **a** Assembly of four SDH subunits (SDHA-D) into functional SDH and its position in the mitochondrial inner membrane (IMM) facing matrix. The catalytic subunit SDHA contains the flavin cofactor FAD which accepts electrons from succinate and passes them to FeS centres in the SDHB subunit. The electrons are then accepted by membrane carrier ubiquinone (Q) at the Q binding site comprised by membrane embedded SDHC and SDHD subunits. Reduced Q (QH_2_) transfers electrons within the IMM to complex III. **b** Enzymes of TCA cycle and accompanying replenishing reactions. CS citrate synthase, IDH isocitrate dehydrogenase, OGHD oxoglutarate dehydrogenase, SDH succinate dehydrogenase, FH fumarate hydratase, MDH malate dehydrogenase, PDH pyruvate dehydrogenase, PC pyruvate carboxylase, GOT glutamate-oxaloacetate transaminase, GLDH glutamate dehydrogenase. Enzymes with mutations found in PPGL are outlined in orange. Grey colour depicts pathways important in SDH deficiency (see text for details)

Seventeen years ago, the first germ line mutation associated with this disease was identified in the *SDHD* gene, which encodes for one of four subunits of the enzyme, succinate dehydrogenase enzyme (SDH), also known as mitochondrial complex II (CII) (Baysal et al. 2000) (Fig. 1a). This was a significant finding, as until that date, no direct connection between the metabolic dysfunction and the causation of cancer had been identified. This finding also contributed to the renewed interest in Otto Warburg’s postulate almost 100 years before that defective mitochondrial metabolism was the major cause of cancer development (Koppenol et al. 2011). Over the next few years, mutations in the other three SDH genes (A, B and C) and its assembly factor (SDHAF2) were also observed, adding considerable evidential weight to *SDHx* genes being critical tumour suppressors linked to PPGL (Astuti et al. 2001; Burnichon et al. 2010; Hao et al. 2009; Niemann and Muller 2000). Subsequently, mutations in other genes encoding metabolic enzymes with a role in the tricarboxylic acid cycle (TCA) followed; fumarate hydratase (FH) (Castro-Vega et al. 2014; Letouze et al. 2013) and malate dehydrogenase (*MDH2*) (Cascon et al. 2015) (Fig. 1b). However, it is not only genes encoding metabolic enzymes that are associated with PPGL. Long before the discovery of mutations in *SDHx* in PPGL, the major genetic mutations associated with these cancers were of neurofibromatosis-1 (*NF1*), *RET* and von Hippel-Lindau (*VHL*) (Else 2015). Interestingly, the transcriptional profiling of tumours with mutations in these different genes shows that they segregate into two clusters: a ‘pseudohypoxic’ cluster that includes tumours with *VHL* and *SDHx* mutations and an ‘activated tyrosine kinase’ cluster, originally containing tumours with *RET* and *NF1*(Dahia et al. 2005). More recently, further clustering analyses have been performed that have highlighted new subgroups (Fishbein et al. 2017), but the pseudohypoxic grouping based around SDH and VHL mutations remains a major phenotypically characterised cluster.

## Metabolic alterations in cells with mutations in TCA cycle enzymes

Since cancer is characterised by an unrestricted growth, it seems counterproductive that deficiencies in apparently essential metabolic enzymes can be oncogenic. However, studies into the metabolic alterations in cells harbouring mutations in TCA cycle enzymes have been held back by the lack of appropriate cell models. Indeed, investigations that have utilised cells with varying degrees of knockdown have yielded conflicting results; most likely due to insufficient knockdown, or perhaps different cell backgrounds. More recently, novel models of FH and *SDHx* deficiency have been created using the Cre-lox system and have provided important information regarding the re-wiring of the metabolic network in response to loss of these key enzymes (Cardaci et al. 2015; Frezza et al. 2011; Lussey-Lepoutre et al. 2015). The fact that the TCA cycle is actually not a true cycle, but that its metabolites are replenished or used up by various other interconnected pathways, very probably is the key to cell survival in the absence of these enzymes. However, it remains a critical unanswered question as to why these tumours only arise in certain cell types—suggesting that some of the metabolic re-wiring required for survival is inconsistent with viability in most differentiated cell types.

The mechanism of survival of FH-deficient cells was originally elucidated by Frezza and colleagues using *Fh1*-deficient epithelial kidney cell lines in 2011 and showed that the haem biosynthetic pathway became essential in these conditions (Frezza et al. 2011). They found that the TCA cycle truncation at FH led to an accumulation of mainly glutamine-derived fumarate (and to some degree, succinate), but low malate and citrate levels. Interestingly, although mitochondrial respiration was decreased, *Fh1*-deficient cells still retained normal mitochondrial membrane potential and were capable of generating and oxidising NADH. This NADH was very probably produced by glutamate metabolism through glutamate and 2-oxoglutarate (2OG) dehydrogenases (see Fig. 1b brown dashed lines). However, this critical metabolic activity could only be maintained through a metabolic ‘escape route’—the haem biosynthesis pathway. This pathway, which was found to be essential in FH-deficient cells, metabolises succinyl-CoA to synthesise haem, but in this case, further metabolises the haem to form bilirubin, which is excreted (Frezza et al. 2011). Further, metabolic re-wiring was observed in these cells that impinged on urea cycle activity (Adam et al. 2013; Zheng et al. 2013). FH-deficient cells have been shown to metabolise fumarate through reversal of the activity of argininosuccinate lyase, using arginine and accumulated fumarate to produce argininosuccinate, which they also excrete (Adam et al. 2013; Zheng et al. 2013).

Two recent studies on metabolic re-wiring in SDH-deficient cells have shown that a seemingly similar metabolic deficiency results in an significantly different metabolic phenotype, with cells dependent on pyruvate carboxylase (PC) activity for aspartate synthesis (Cardaci et al. 2015; Lussey-Lepoutre et al. 2015) (see Fig. 1b grey dashed lines). Additionally, it was observed that SDH-deficient cells exhibit perturbed redox homeostasis—evidenced by increased pyruvate reduction to lactate (Cardaci et al. 2015). This is likely a consequence of altered TCA cycle activity, but the precise nature of the change in redox function within SDH-deficient cells is still unclear, with a further study suggesting that *SDHx* mutations that are oncogenic differ from those associated with neurodegenerative diseases in that only the former result in decreased NADH oxidation by complex I of the respiratory chain (Lorendeau et al. 2017). It is interesting that neither Cardaci et al. nor Lussey-Lepoutre et al. presented data regarding whether deficiencies in SDH lead to an increase in the haem biosynthesis pathway, reminiscent of FH-deficient cells (Cardaci et al. 2015; Lussey-Lepoutre et al. 2015). Clearly, this is an attractive hypothesis, and the lack of data presented suggests that the results from their studies were not as clear-cut as expected. It appears therefore that our understanding of the metabolic consequences of apparent loss of function mutations within the subunits of SDH remains very much incomplete, and further studies are warranted.

## Metabolic signalling in pseudohypoxic systems

In addition to the metabolic re-wiring that occurs as a consequence of loss of function of TCA cycle enzymes, the metabolites that accumulate have also been shown to act as signalling molecules, directly inducing malignant phenotypes. Many TCA cycle metabolites can serve as a signal influencing various cellular processes (Frezza 2017), but 2OG, succinate and fumarate stand out when it comes to hypoxic and pseudohypoxic signalling with a profound impact on cancer evolution and progression (Morin et al. 2014; Sullivan et al. 2016).

One of the most important longer-term physiological responses to hypoxia is the increase in red blood cell production to aid oxygen delivery to peripheral tissues (Weir et al. 2005). The mechanism by which this occurs was first described in 1992, involving hypoxia-induced binding of the transcription factor, hypoxia-inducible factor 1 (HIF1), to the promoter of the erythropoietin gene (Semenza and Wang 1992). In the same year, vascular endothelial growth factor (VEGF) was shown to mediate hypoxia-induced angiogenesis (Plate et al. 1992; Shweiki et al. 1992) and was subsequently also confirmed as a HIF1 target gene (Forsythe et al. 1996). Since then, HIF1 has been shown to regulate the expression of hundreds of genes, including those encoding for metabolic enzymes (e.g. lactate dehydrogenase A and pyruvate dehydrogenase kinase 1), angiogenic factors, extracellular matrix remodelling enzymes and cell cycle regulating factors (Pugh and Ratcliffe 2017).

**Fig. 2:**
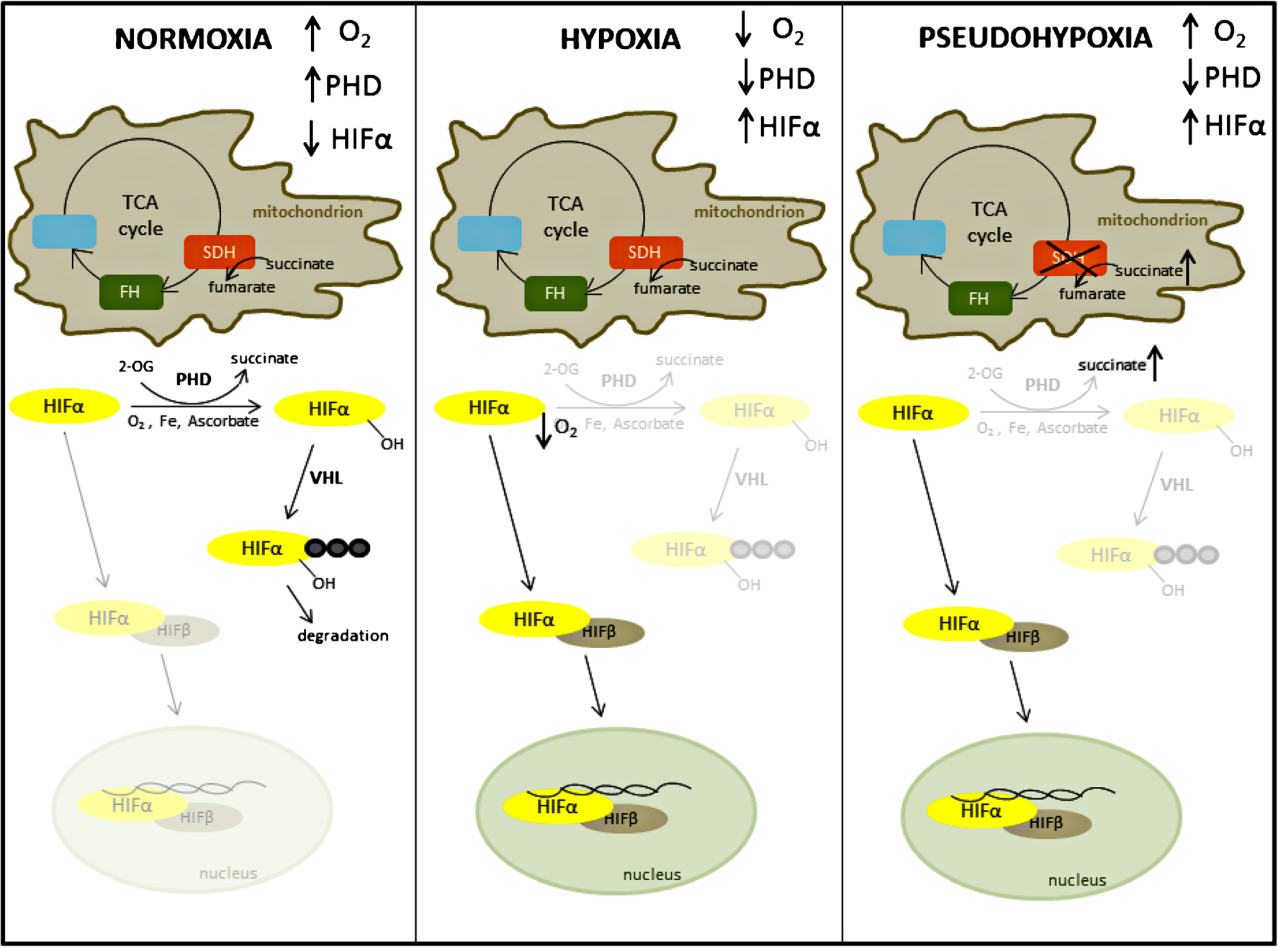
HIF degradation pathway in normoxia and its stabilisation in hypoxia and pseudohypoxia. Under normoxic oxygen levels, PHD enzymes hydroxylate HIFα subunit on specific proline residues. These are recognised by VHL ubiquitin ligase which targets HIFα for proteasomal degradation (first panel). Under conditions of low oxygen levels, PHD enzymes are inhibited and HIFα protein is stabilised, binds HIFβ subunit and transactivates HIF responsive genes (middle panel). In pseudohypoxic conditions depicted here by SDH deficiency, high succinate levels restrict the PHD reaction by product inhibition and HIFα is stabilised under conditions of normoxic oxygen levels (last panel)

The hypoxia-inducible factor (HIF) transcriptional factors are formed of two constitutively expressed subunits—alpha and beta (the latter originally named the aryl hydrocarbon receptor nuclear translocator (ARNT)) (Wood et al. 1996). Oxygen sensitivity is achieved through the rapid post-translational modification and degradation of the α subunit in normoxia, which means that in these conditions, no active HIF heterodimer can be formed (Fig. 2). There are three α subunits, two of which form active transcription factors, known as HIF1 and HIF2 (HIF2α originally known as endothelial PAS domain protein 1) (Tian et al. 1997). The function of both factors is regulated by the action of three oxygen-dependent prolyl 4-hydrogenase (PHDs1-3) that hydroxylate HIFα subunits on specific prolyl residues, thereby forming a recognition site for the von Hippel-Lindau (pVHL)-containing E3 ligase complex, which binds and induces the polyubiquitylation of the HIFα subunits, leading to their proteasomal degradation. Loss of pVHL expression in some PPGL therefore elicits a pseudohypoxic phenotype through the inappropriate stabilisation of HIFα subunits in normoxia. The hydroxylation reaction performed by the PHD enzymes requires oxygen and a metabolic intermediate—2-oxoglutarate—as substrates, as well as ferrous iron and ascorbate as cofactors (Ploumakis and Coleman 2015). Of the three PHD enzymes that are known to hydroxylate the HIFα subunits, PHD2 is thought to be the major regulator of HIF1α expression, while PHD1 and 3 may have a preference for HIF2α (Bruick and McKnight 2001; Ivan and Kaelin Jr. 2017; Taniguchi et al. 2013). Additionally, the PHDs are thought to have non-HIF targets, with PHD3 playing a role in regulation of apoptosis (Lee et al. 2005; Tennant and Gottlieb 2010) and metabolism (German et al. 2016; Luo et al. 2011). Although the PHD enzymes control HIFα stabilisation, a further level of oxygen and 2OG-mediated control is provided by factor inhibiting HIF (FIH), which hydroxylates HIFα on an asparaginyl residue in the C-terminal transactivation domain (CAD) abrogating its interaction with p300, thereby preventing transcriptional activation (Hewitson et al. 2002).

PHD and FIH enzymes that target HIFα subunits are not the only 2OG-dependent dioxygenases but belong to a large family of more than 70 enzymes (Ploumakis and Coleman 2015). These include important demethylases that modulate the epigenome and in this way can affect multiple cellular pathways and play a prominent role, particularly in cancer. Recently, it was shown that ten-eleven translocation (TET) DNA hydroxylases, which demethylate genomic DNA in a multistep process initiated by hydroxylation of 5-methylcytosine, manifest reduced activity by tumour hypoxia in human and mouse cells and result in hypermethylation of the tumour suppressor gene promoters. Interestingly, the authors provide evidence that this change in TET activity depends directly on availability of oxygen (Thienpont et al. 2016).

The first association of increased concentrations of metabolites and the pseudohypoxic phenotype was revealed in 2005 when the link was provided between loss of SDH function, increased succinate levels and the resulting inhibition of 2OG-dependent PHD enzymes and stabilisation of HIFα subunits under normoxic conditions (Selak et al. 2005). Increased levels of succinate in tumour samples from PPGL patients have been confirmed in a number of studies (Imperiale et al. 2015; Lehtonen et al. 2007; Lendvai et al. 2014; Pollard et al. 2005; Richter et al. 2014) and high succinate to fumarate ratios suggested as a metabolic marker for the detection of SDHB/SDHD-related PPGL tumours (Lendvai et al. 2014). As the hydroxylation of HIFα by PHDs oxidatively decarboxylates 2OG to form succinate (Ploumakis and Coleman 2015), at least some of the effect of succinate is likely through product inhibition of the PHDs (see Fig. 2). Similarly to high succinate levels, fumarate has also been shown to inhibit PHDs and stabilise HIFα when FH was inactivated (Isaacs et al. 2005). In contrast to PHDs, the other HIFα-acting hydroxylase, FIH (factor inhibiting HIF), appears relatively insensitive to inhibition by succinate or fumarate accumulation (Koivunen et al. 2007). The effect of another more recently described 2OG-like metabolite, 2-hydroxyglutarate (2-HG), which arises as the result of oncogenic isocitrate dehydrogenase (IDH) mutations, is not yet clear, seemingly dependent on the 2OG-dioxygenase enzyme involved. D-2-HG can increase or decrease the activity of PHDs, while the L-2-HG has been reported to competitively inhibit PHD enzymes (Sullivan et al. 2016), although this may be through non-enzymatic oxidation to 2OG (Tarhonskaya et al. 2014). Recently, other means of inhibiting PHD enzymes have been suggested, including deficiencies in the TCA cycle enzyme, 2OG dehydrogenase (Burr et al. 2016) and depletion of intracellular cysteine (Briggs et al. 2016). With respect to non-HIF-mediated mechanisms of pseudohypoxia, it was recently shown that fumarate accumulation can drive a number of additional HIF-independent phenotypes, including TANK-binding kinase 1 (TBK1)-mediated activation of nuclear factor kappa-light-chain-enhancer of activated B cell (NFκB) signalling (Shanmugasundaram et al. 2014) and succination of an steadily increasing number of proteins (Blatnik et al. 2008; Kinch et al. 2011; Miglio et al. 2016; Ternette et al. 2013; Yang et al. 2014).

Interestingly, the pseudohypoxic cluster of PPGL associated with *SDHx* and *VHL* mutations can be further stratified using the DNA methylation profile (Letouze et al. 2013). The ‘hypermethylator’ phenotype of *SDHx*-mutated tumours is a product of succinate-dependent inhibition of the TET family of DNA demethylases (Killian et al. 2013; Kiss et al. 2008; Letouze et al. 2013; Xiao et al. 2012), hence is not observed in *VHL*-mutated disease. Interestingly, tumours harbouring mutations in FH were also found to have hypermethylated DNA similarly to the SDHB tumours, in which the epigenetic silencing was particularly severe compared to those tumours with mutations in other SDH subunits (Castro-Vega et al. 2014; Letouze et al. 2013). It was proposed that SDH inactivation may be more complete in SDHB-mutated tumours vs. tumours with mutations in other SDH subunits resulting in a stronger inhibition of 2-OG-dependent demethylation due to higher succinate levels, but the authors were unable to confirm this hypothesis experimentally (Letouze et al. 2013).

## HIF association with cancer and PPGL

As already mentioned in the ‘Introduction’, there are a number of means by which HIF signalling is beneficial for tumours. However, although HIF1 and HIF2 share many common features, the timing and regulation of their stabilisation and differences in the genes they control mean that their relative expression, which could be influenced by factors including oxygen tension and cellular metabolism, will significantly influence the final hypoxic phenotype (Keith et al. 2011). This is also likely true of pseudohypoxia. Although HIF1α is ubiquitously expressed, HIF2α was first thought to be endothelial cell specific. However, it has now been confirmed in non-endothelial cells of various tissues, including brain, heart, lung, kidney, liver, pancreas and intestine (Wiesener et al. 2003). Further complexity lies within the nature of the genes whose expression is altered by the different HIF transcription factors. An example of this is that HIF1 activity increases expression of enzymes that control glucose metabolism, while HIF2 can drive de-differentiation through increasing expression of proteins such as SOX2, NANOG and OCT4—well-described stem cell markers (Covello et al. 2006; Lee et al. 2016b). A final way in which the HIF transcription factors may produce divergent phenotypes is through their interaction with and control by other cancer-associated factors, such as p53, mTOR and MYC (Keith et al. 2011). In contrast to HIF1, HIF2 has been suggested to inhibit p53 and stimulate mTORC1 to promote proliferation in hypoxia. HIF1 also is thought to disrupt MYC-dependent gene transactivation, while HIF2 collaborates with MYC to promote its oncogenic activities (Keith et al. 2011).

Besides the benefits of neovascularisation driven by HIF-dependent VEGF expression, HIF also orchestrates a metastatic transcriptome, including downregulation of the intercellular adhesion molecule E-cadherin (Esteban et al. 2006) and degradation of extracellular matrix (Krishnamachary et al. 2003). HIF1 also promotes genome instability through suppression of DNA repair pathways and inhibition of DNA mismatch repair gene (Bristow and Hill 2008). Both HIF1 and HIF2 induce chemoresistance by increasing expression of drug efflux pumps from the ATP-binding cassette transporter family (Comerford et al. 2002; Martin et al. 2008), and they are involved in the hypoxia-induced resistance to radiotherapy (Bertout et al. 2009; Harada et al. 2012). Importantly, the HIF transcription factors are master regulators of a (pseudo)hypoxia-induced metabolic reprogramming that induces increased glucose metabolism (a Warburg phenotype, when in pseudohypoxia), suppresses glucose oxidation in the mitochondria and supports synthesis of various macromolecules and building blocks needed for constant DNA replication and cellular growth (Vander Heiden et al. 2009).

In most tumours, hypoxia and pseudohypoxia may represent a consequence of cancer development and progression. However, in the pseudohypoxic cluster of PPGL, the pseudohypoxic phenotype is likely causal (Amorim-Pires et al. 2016). Increased stabilisation of both HIF1 and 2 have been reported in PPGL tissues (Favier et al. 2009; Lopez-Jimenez et al. 2010; Pollard et al. 2005; Pollard et al. 2006), but consistently with other pseudohypoxic tumours, the role of HIF2 may be more important and widespread (Comino-Mendez et al. 2013; Welander et al. 2014). Since the first report of an *EPAS1* (HIF2α) mutation in PPGL in 2012 (Zhuang et al. 2012), a number of studies have demonstrated *EPAS1* mutations, with an overall frequency suggested as between 6 and 12% (Toledo 2017).

Although *VHL* and *SDHx*-mutated PPGLs cluster together on the basis of their pseudohypoxic phenotype (Dahia et al. 2005), differences in the precise nature of their pseudohypoxic signature have been reported (Favier et al. 2009; Lopez-Jimenez et al. 2010), with strong activation of HIF1 target genes in *VHL*-mutated PPGL (Burnichon et al. 2016; Lopez-Jimenez et al. 2010). A higher mRNA expression of the HIF1 (but not HIF2) target genes, GLUT1 and HK2, was reported in *VHL*-mutated compared to *SDHB*-mutated adrenal medulla tissue (Fliedner et al. 2012), and *VHL*-mutated tumours demonstrate increased glycolysis in comparison to *SDHx*-derived PPGL (Favier et al. 2009). Interestingly, though strong HIF2α staining has been shown in both *VHL*-mutated and *SDH*-mutated tumours (Favier et al. 2009; Lopez-Jimenez et al. 2010), PPGL with HIFα mutations demonstrate a different pseudohypoxic transcriptome, suggesting that a further transcriptional driver is involved (Fliedner et al. 2016). Consistent with this, there is evidence that activation of HIFs may be important, but not sufficient, for the fully malignant phenotype of pseudohypoxic tumours. In a mouse HLRCC mode with renal tubule-specific *Fh1*^*−/−*^, genetic inactivation of HIF1α or HIF2α, either alone or in combination, failed to ameliorate the development of renal cysts (Adam et al. 2011). Indeed, as the cystic phenotype was exacerbated in the *Fh1*^*−/−*^/*Hif1*^*−/−*^ model, it was suggested that in this system, it may be tumour suppressive (Adam et al. 2011). Hence, although the role of HIF2 as driving malignancy is clear, the role of HIF1 is less so and may be instead supportive of oncogenic transformation.

## The role of reactive oxygen species in hypoxia and pseudohypoxia

**Fig. 3:**
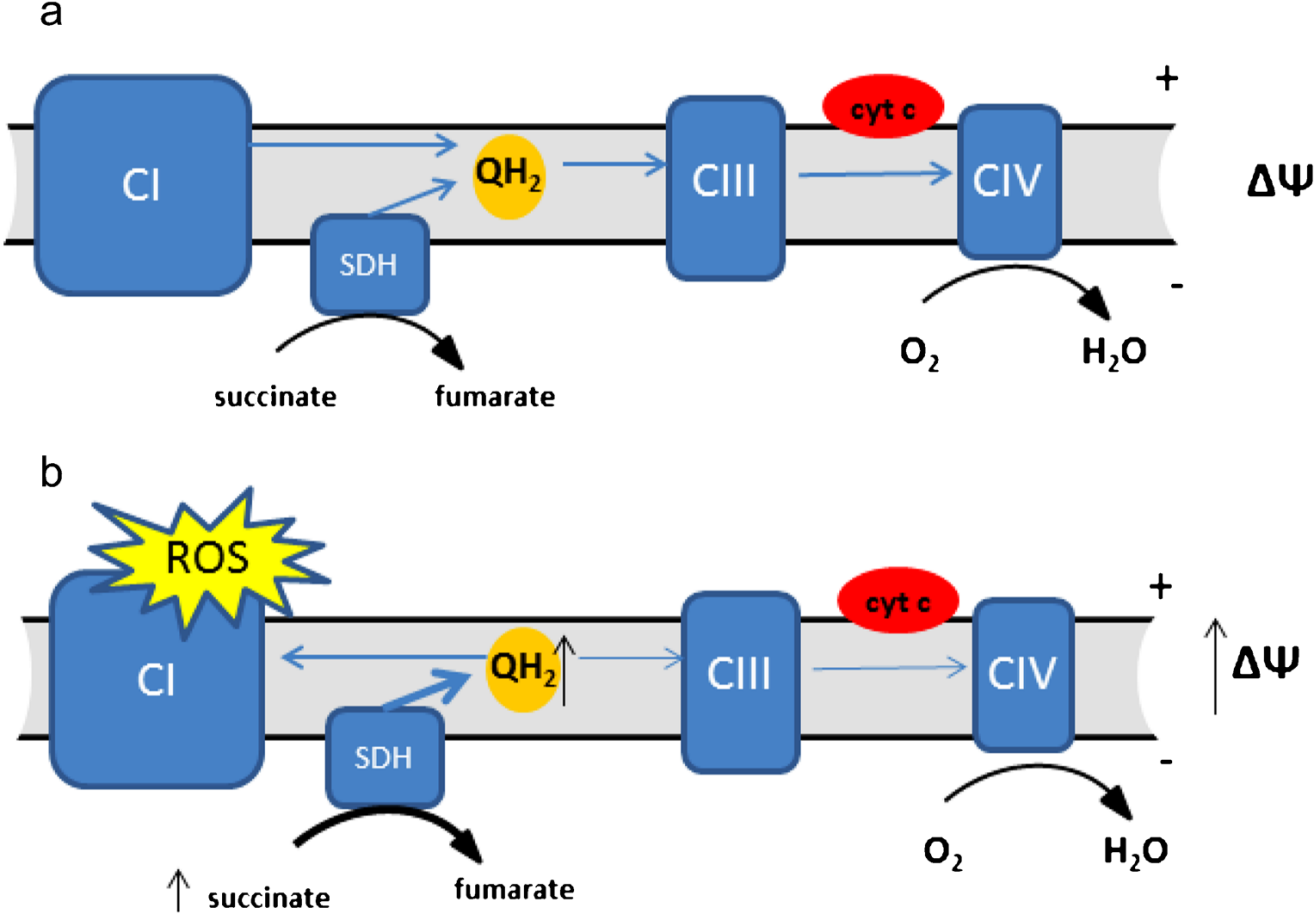
Electron transport chain and reverse electron transfer. **a** Membrane carrier ubiquinone accepts electrons from CI and SDH (QH_2_) and transports them to CIII from where the electrons are carried by cytochrome c (cyt c) to CIV and reduce molecular oxygen to water. **b** Under conditions of high concentrations of succinate and high mitochondrial transmembrane potential (ΔΨ), the ubiquinone pool becomes over-reduced and transfers electrons to CI where they escape as ROS

Reactive oxygen species (ROS) are damaging molecules containing oxygen with an unpaired (free) electron that are capable of oxidising cellular macromolecules (Lambert and Brand 2009). The reduction of molecular oxygen with a single electron forms superoxide, which is highly reactive, but with limited permeation through the cell. Superoxide can, however, be reduced to form hydrogen peroxide, a less reactive ROS, which may be able to travel further in the cell, damaging more distant cellular components (Murphy 2009). This effect, though, is off-set through the activity of cellular peroxiredoxin enzymes, which are present at high levels both in the mitochondria and in the cytosol and represent an extremely effective means of detoxifying hydrogen peroxide (Cox et al. 2009). It is a distinct possibility that the localisation of the mitochondria within cells—whether perinuclear or more peripheral—could influence the cellular components that are damaged. A direct role for hydrogen peroxide is therefore unclear. Although ROS are critical for normal cell function, they are also responsible for the oxidative damage observed in pathologies such as neurodegenerative diseases and cancer. Indeed, the oxidative damage of DNA by ROS is thought to be a significant driver of genome instability and therefore mutational load in tumours (Jackson et al. 1998; Kruk and Aboul-Enein 2017). Environments that increase ROS production are therefore likely to be pro-tumourigenic—a hypothesis supported by studies of the effect of environmental oxygen on tumour initiation (Sung et al. 2011). In most cell types, the mitochondria are the major source of ROS, having at least 10 known sites capable of ROS generation, including the electron transport chain (ETC) (Lambert and Brand 2009) of which SDH is an integral part (Fig. 3a).

Though complexes I and III are considered the major ROS producing sites in the ETC, recent studies have shown that under certain circumstances, SDH can also produce a significant amount of ROS (Kluckova et al. 2015; Quinlan et al. 2012) suggested to approach the levels produced by complex III when the redox state of the ETC is suboptimal (Quinlan et al. 2012). It is therefore important to note that excessive ROS levels have been shown to stabilise HIFα regardless of oxygen tension (Guzy et al. 2005), and it has been suggested that this mechanism, through an intact ETC, may be involved in hypoxia-mediated HIFα subunit stabilisation (Chandel et al. 1998). Downregulation of ETC components by either silencing a subunit of complex III or removing cytochrome c has been shown to attenuate HIF1α stabilisation under hypoxia, but retained the ability to stabilise HIF when challenged with direct PHD enzyme inhibitors, such as iron chelators (Brunelle et al. 2005; Guzy et al. 2005; Mansfield et al. 2005). Increased ROS production from the ETC has been suggested to contribute to HIF stabilisation and induction of pseudohypoxia in SDH-mutated PPGL (Guzy et al. 2008). Considering the necessity of the ROS signal for HIF stabilisation under normoxic or hypoxic conditions (Lee et al. 2016a; Waypa et al. 2016), the possibility of increased ROS signalling in SDH-derived tumours has to be considered. However, the experimental evidence for increased ROS in various models of SDH dysfunction is not consistent, with evidence suggesting that they are increased (Guzy et al. 2008; Ishii et al. 2005; Saito et al. 2016) or normal (Cervera et al. 2008; Selak et al. 2005). The authors in the mentioned studies chose to genetically manipulate different SDH subunits of which all impaired SDH activity to various levels (but none of which resulted in complete loss of activity, as observed in patient tumours), and despite the disparate observations regarding ROS production, HIF stabilisation was observed in all models whether higher ROS were detected or not. As mentioned previously, HIF stabilisation in SDH dysfunctional cells is expected through non-ROS mechanisms, as increased succinate levels are known to inhibit the PHD enzymes, and therefore, a thorough examination of the potential role of ROS, independently of other factors, is still required. A study by Guzy and colleagues reported increased ROS in cells stably silenced for SDHB, and this ROS production was suppressed by addition of the SDHA site inhibitor. While SDHB knockdown induced ROS and stabilised HIF, SDHA knockdown did not (Guzy et al. 2008). Further, the authors showed that pharmacological inhibition of the ubiquinone-binding site formed by SDHC/SDHD subunits resulted in ROS production and HIF stabilisation. This study therefore provided experimental proof for the production of ROS in PPGL arising from SDHB-D mutations. It is possible that tumours founded upon SDHA mutations, which were only characterised in 2010 (Burnichon et al. 2010), may be different in terms of their ROS production. Contrary to Guzy’s observations of ROS production after SDHB downregulation, Cervera et al. were unable to detect measureable changes in ROS levels, and by assessing this in cells silenced for SDHB either transiently, stably or by expression of missense mutant SDHB genes, these authors were unable to support a hypothesis that alterations in SDHB activity could result in increased ROS production (Cervera et al. 2008). Interestingly, among other cell models used, both studies used human hepatoma Hep3B cell line but different ROS detection strategies. Also throughout the other studies mentioned, various ROS detection techniques were used and often more approaches to validate the results obtained with one detection system, but within one study, various detection methods yielded similar results, so it is not easy to ascribe the inconsistency in ROS observations to variability in the detection systems.

Important direct evidence for the interplay between succinate and ROS production by mitochondria arose from the studies of isolated mitochondria, which showed that high concentrations of succinate elicit very high ROS production (as discussed in Lambert and Brand (2009)). These ROS were found to be due to a reverse electron transfer (RET) from an over-reduced ubiquinone pool which lies between complex I/II and complex III within the ETC (Lambert and Brand 2009) (Fig. 3b). This effect was confirmed in a physiologically relevant model of cardiac or brain ischemia/reperfusion injury, where RET is the result of hypoxic succinate accumulation in affected tissues and high SDH activity occurred during subsequent reperfusion (Chouchani et al. 2014).

Important evidence for the role of mitochondrial regulation in paragangliomas can be observed in the normal function of the carotid body—a common site for these tumours to form. In the glomus cells of this tissue, functional complex I of the ETC was suggested as a necessary requirement for oxygen sensing (Fernandez-Aguera et al. 2015) through the study of complex I-deficient (*Ndufs2*^*−/−*^) mice. In these mice loss of responsiveness to hypoxia was observed, which was suggested to be due to loss of a required ROS signal from complex I. Interestingly, the authors noted high SDH activity in glomus cells in wild-type mice, which was accompanied by high levels of succinate, suggestive of RET and ROS production (Fernandez-Aguera et al. 2015). It is therefore highly relevant in the SDH-deficient model as to the nature of SDH inactivation, and whether complex I is inactivated as previously suggested (Lorendeau et al. 2017), as this may represent a reason for not only some of the discrepancies in experimental findings about ROS production in these models, but also the biological outcomes of loss of SDH function. In the context of pheochromocytomas, if RET from high succinate via complex II is involved in the oxygen sensing, SDH mutations should impair the organismal hypoxia response and would be expected to result in the abolition of catecholamine secretion through loss of ROS-mediated K^+^ channel modulation. However, the opposite is true—patients do not lose ability to synthesise catecholamines (Zuber et al. 2011). Additional evidence arises from studies of mice heterozygous for *Sdhd*, which despite being haploinsufficient show persistent carotid body glomus cell activation and full responsiveness to hypoxia which suggests that complex II is probably not directly involved in carotid body oxygen sensing (Piruat et al. 2004). Interestingly, no tumours were observed in these mice, nor in those with catecholaminergic tissue-specific *Sdhd*^*−/−*^ (Diaz-Castro et al. 2012; Piruat et al. 2004).

## Conclusions/summary

Mutations in SDH (and other TCA cycle enzymes) are oncogenic, forming tumours in a select subset of tissues. The reason for this tissue selectivity remains unknown—something that is not helped by the fact that despite continued attempts, mouse models driven by loss of these enzymes do not appear to form tumours (Diaz-Castro et al. 2012; Lepoutre-Lussey et al. 2016; Macias et al. 2014; Pollard et al. 2007; Rankin et al. 2006). Although *VHL*-driven tumour models have been achieved, this was possible by mutating further tumour suppressor genes (Bailey et al. 2017; Harlander et al. 2017). It is therefore probable that in common with this, additional mutations are required in addition to those in SDH subunits to reproduce the human disease in mice (Adam et al. 2014). Consistent with this, mutations in the genes encoding ATRX (involved in telomere maintenance) and in the promoter region of telomerase itself (TERT) have been reported in SDH-deficient tumours (Fishbein et al. 2015; Papathomas et al. 2014). Interestingly, with the advent of next generation sequencing technologies, increasing numbers of metabolic enzymes are being suggested as drivers of PPGL (Castro-Vega et al. 2014; Toledo et al. 2013). Although this introduces an apparent increase in complexity to the field, it is likely that these mutations will greatly help our understanding of the metabolic changes required for transformation—whether this is increased succinate concentrations, changes in redox homeostasis, ROS production, the pseudohypoxic signalling cascades observed or indeed more complex metabolic changes. Indeed, it is even possible that increased succinate concentrations are not the oncogenic driver after all.

Given the range of mutations observed in PPGL represented by *VHL*, *EGLN1* (PHD2) and *EPAS1* (HIF2α) of which all directly control hypoxic signalling and with SDH and FH mutations further providing a link to hypoxia, pseudohypoxia seems to be an undeniable feature of PPGL. Intriguingly, pseudohypoxic HIF signalling has also been suggested as an important driver in the tumourigenesis of tumours with mutations observed in cluster 2 PPGL, as their signalling via Ras/MAPK, PI3K/AKT and mTORC pathways could result in increased HIF signalling (Jochmanova et al. 2013). Increased activity of these pathways is very common in many different cancer types and has been shown to upregulate HIF, but this is yet to be demonstrated in cluster 2 PPGL, indeed, the fact that they are cluster 2 is a de facto demonstration that they differ significantly from the pseudohypoxic cluster (Dahia et al. 2005; Fishbein et al. 2017; Lopez-Jimenez et al. 2010). Nevertheless, as the links between hypoxia and HIF stabilisation in PPGL cancers are too strong, it remains something to be considered—especially given the ongoing clinical trials evaluating agents that target drivers and effectors of the pseudohypoxic phenotype: the VEGFA receptor and HIF2α (Toledo 2017). We are in a highly exciting time for this rapidly evolving field, where novel technologies are playing a vital role in revolutionising our understanding of the biology of these tumours. The outlook for patients with these tumours is therefore improving and will continue to do so as new findings are translated into novel therapies.
